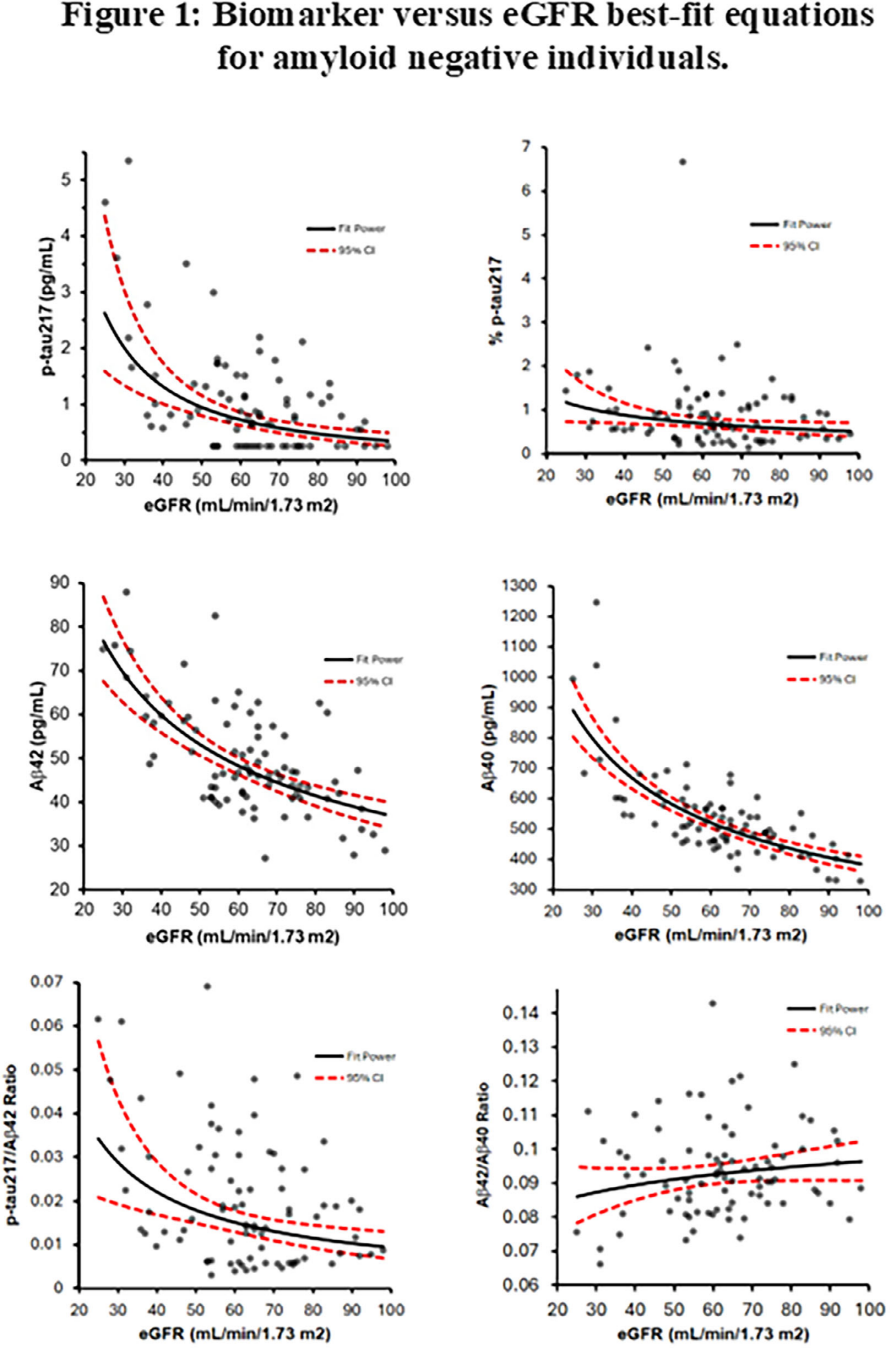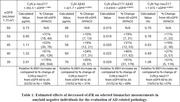# Comparison of the Effect of Chronic Kidney Disease on Plasma *p*‐tau217 Determinations versus %p‐tau217 and the *p*‐tau217/Aβ42 Ratio in an Amyloid Negative Cohort

**DOI:** 10.1002/alz70856_101203

**Published:** 2025-12-24

**Authors:** Joshua A Bornhorst, Chelsea Swartchick, Prashanthi Vemuri, Jonathan Graff‐Radford, David S. Knopman, Stephen D. Weigand, Michael E. Griswold, Petrice M Cogswell, Clifford R. Jack, Ronald Petersen, Alicia Algeciras‐Schimnich

**Affiliations:** ^1^ Mayo Clinic, Rochester, MN, USA; ^2^ Department of Radiology, Mayo Clinic, Rochester, MN, USA; ^3^ Department of Neurology, Mayo Clinic, Rochester, MN, USA; ^4^ University of Mississippi Medical Center, The MIND Center, Jackson, MS, USA

## Abstract

**Background:**

Chronic kidney disease (CKD) is associated with increased blood‐based biomarker concentrations, potentially confounding their utility in Alzheimer's disease (AD) evaluation. While plasma phosphorylated tau217 (*p*‐tau217) is elevated in the presence of amyloid related pathology, amyloid beta 1‐42 (Aβ42) is decreased. We investigated the association of plasma *p*‐tau217, non‐phosphorylated tau217 (np‐tau217), Aβ42, Aβ40, as well as the Aβ42/Aβ40 and *p*‐tau217/Aβ42 ratios, versus decreased estimated glomerular filtration rate (eGFR in mL/min/1.73 m^2^) in individuals without amyloid pathology.

**Method:**

This study included participants with CKD stages 1‐2 (eGFR 60‐98, n = 50), and stages 3‐4 (eGFR 25‐59, n = 33), who were Pittsburgh ^11^C‐Compound B amyloid‐PET negative (Centiloid of < 25). Plasma *p*‐tau217, np‐tau217, Aβ42, and Aβ40 were measured by immunoprecipitation mass spectrometry (C_2_N Diagnostics). %p‐tau217 [(*p*‐tau217/np‐tau217) x100], Aβ42/Aβ40 ratio, and *p*‐tau217/Aβ42 ratio were calculated. Log‐Log (power function) best‐fit regression analysis was used to quantitatively predict effects of changes in eGFR (2021 CKD‐EPI equation) versus each single analyte or ratio (Figure 1).

**Result:**

Selected best‐fit analyte equations versus eGFR are shown in Table 1. The predicted percent increase of *p*‐tau217 as eGFR decreases from 60 to 45 was 52% (95% CI: 30–78%). Significantly smaller increases of 16% were predicted for Aβ42 (95% CI: 12–21%), and 19% for Aβ40 (95% CI: 16–23%) over the same eGFR range. The best‐fit predicted increase for the *p*‐tau217/Aβ42 ratio and %p‐tau217 as eGFR decreases from 60 to 45 was 31% (95% CI: 12–53%), and 19% (95% CI: 2–38%), respectively. While not recommended for assessment of amyloid pathology, the Aβ42/Aβ40 ratio did not exhibit a predicted increase (‐2.3% decrease, 95% CI: ‐5.2–0.6%) versus decreasing eGFR from 60 to 45.

**Conclusion:**

The use of either %p‐tau217 or the *p*‐tau217/Aβ42 ratio (to a lesser degree) reduced the confounding effects of decreasing eGFR as compared to *p*‐tau217 alone. Aβ42 and Aβ40 exhibited similar reduced predicted increases versus decreasing eGFR as compared to *p*‐tau217. Relative to the predicted effects of decreasing eGFR on *p*‐tau217 measurements, the *p*‐tau217/Aβ42 ratio relative increase was reduced by 40%, while the relative increase was reduced by 63% for %p‐tau217 measurements.